# Supplementary material for: Structure and function of a malaria transmission blocking vaccine targeting Pfs230 and Pfs230-Pfs48/45 proteins
Source: Commun Biol. 2020 Jul 24;3:395. doi: 10.1038/s42003-020-01123-9 (PMC7381611; doi:10.1038/s42003-020-01123-9)
Supplement: Supplementary file 1 — Supplementary Information [file 42003_2020_1123_MOESM1_ESM.pdf]

Structure and function of a malaria transmission blocking vaccine targeting Pfs230 and Pfs230-Pfs48/45 proteins

Supplementary Figures

Supplementary Tables

SUPPLEMENTARY FIGURES

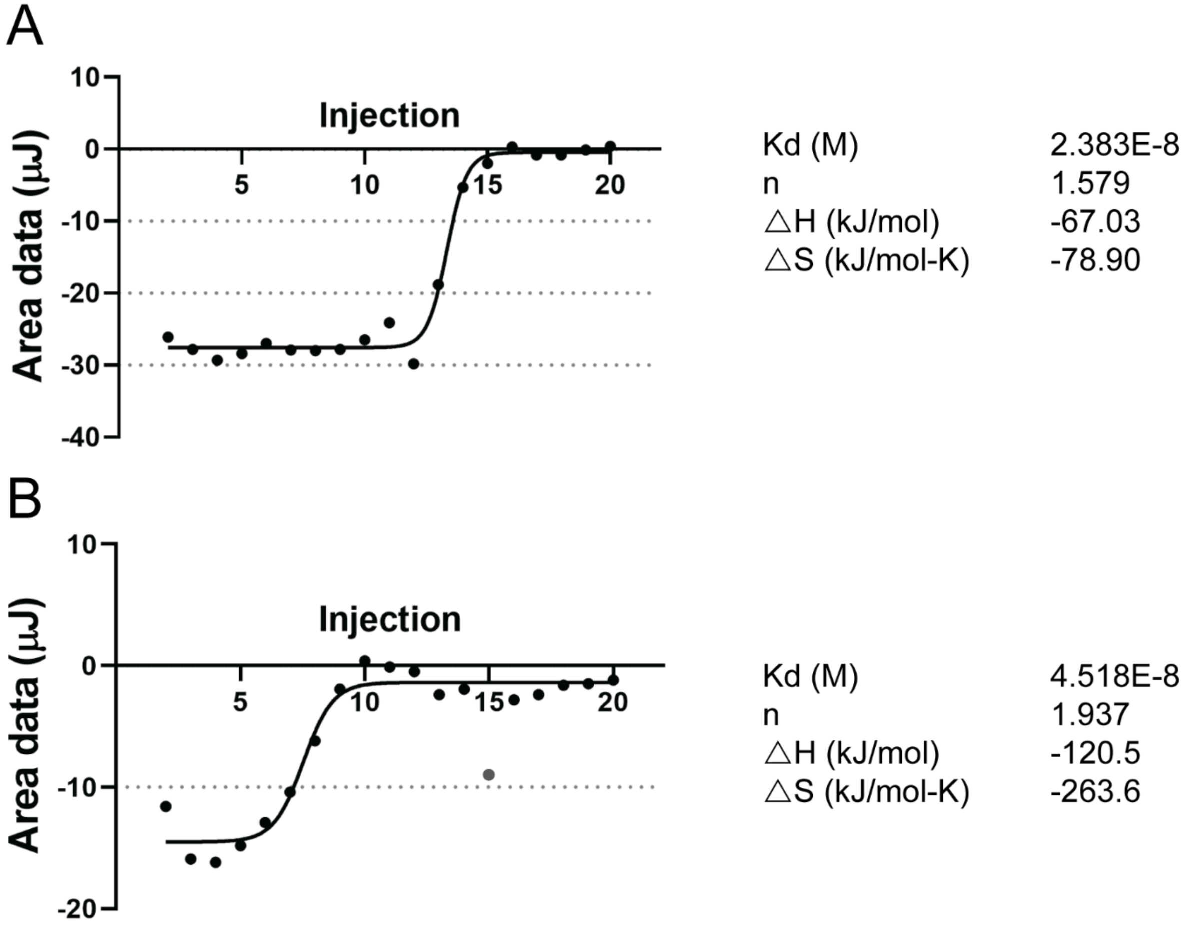

Supplementary Fig. 1. Evaluation of Pfs230D1M (A) and Pfs230D1A (B) binding to mAb 4F12 by isothermal titration calorimetry (ITC). The curve was fitted to the enthalpies of each injection (*black circles*) using an independent binding site model as implemented in the TA Instruments NanoAnalyze software. The insets show the values of the dissociation constant K_d, binding stoichiometry n, the enthalpy ΔH, and entropy ΔS of binding. Binding experiments were performed on a TA Instruments Low Volume Nano ITC by using 20 incremental titrations of 2.5 μL each at 25 °C. The solution containing Pfs230D1M and Pfs230D1A were loaded in the syringe at 165 μM and 49 μM concentrations, and the concentration of 4F12 in the calorimeter cell was 23 μM and 3 μM, respectively. The datum observed for Pfs230D1A injection 15 (*panel B, gray circle*) was an outlier and removed from the analysis.

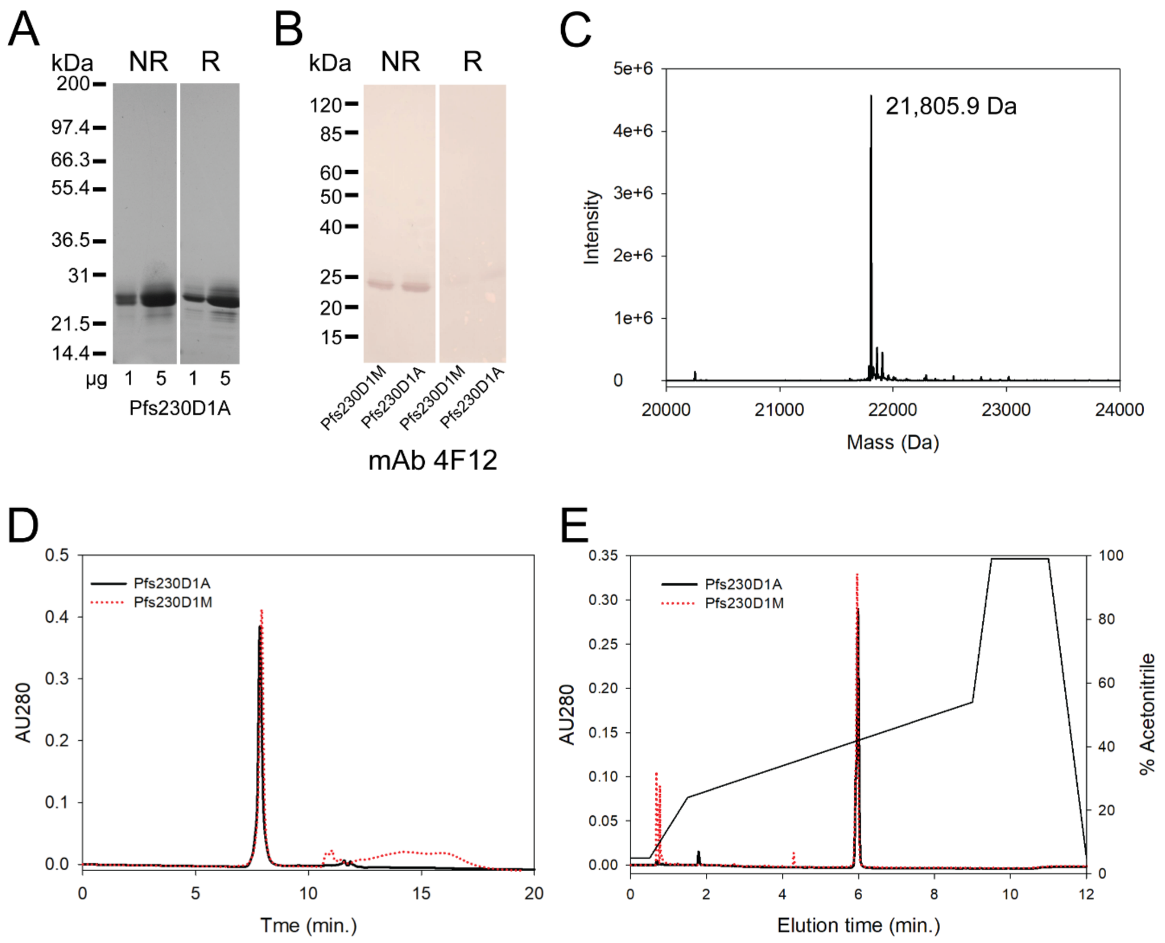

Supplementary Fig. 2. Production of recombinant Pfs230D1A. (A - E) the biochemical and biophysical characterizations of Pfs230D1A. (A) Coomassie blue stained SDS-PAGE analysis of recombinant Pfs230D1A under non-reduced (NR) and reduced (R) conditions. (B) Western blot analysis with Pfs230D1 specific mAb 4F12 against Pfs230D1A and Pfs230D1M under NR and R conditions. (C) Analysis of Pfs230D1A intact mass by LC/MS. (D & E) Overlays of a comparative analysis of Pfs230D1A (black lines) and Pfs230DM (red lines) by SEC-UPLC and RP-UPLC, respectively.

The recombinant Pfs230D1A protein is identical to Pfs230D1M except for Q585 which was changed to N585 and of T587 which was mutated to A587 to retain the disruption of the predicted N-linked glycosylation site. The purified recombinant Pfs230D1A had the expected N-terminus (SVLQSGAL) and a native C-terminus. An analysis of the purified protein by Coomassie blue stained SDS-PAGE and Western blotting with Pfs230D1 specific mAb 4F12 demonstrate the purity and mAb 4F12 reactivity (Supplemental Fig. 2 A & B, respectively). The expected intact mass (21,805.8 Da) was consistent with the observed intact mass (21,805.9 Da) (Supplemental Fig. 2 C). An expected shift in the intact mass of approximately 4 Da was observed upon reduction of Pfs230D1A (21,810 Da), consistent with the presence of two disulfide bonds. An analysis of the purity and integrity of Pfs230D1A in comparison with Pfs230D1M by analytical size exclusion chromatography-UPLC (SEC-UPLC) and by reversed phase-UPLC (RP-UPLC) showed single overlapping peaks. The differences in the SEC-UPLC baselines post-protein elution (D) are due to differences in the buffered protein solutions.

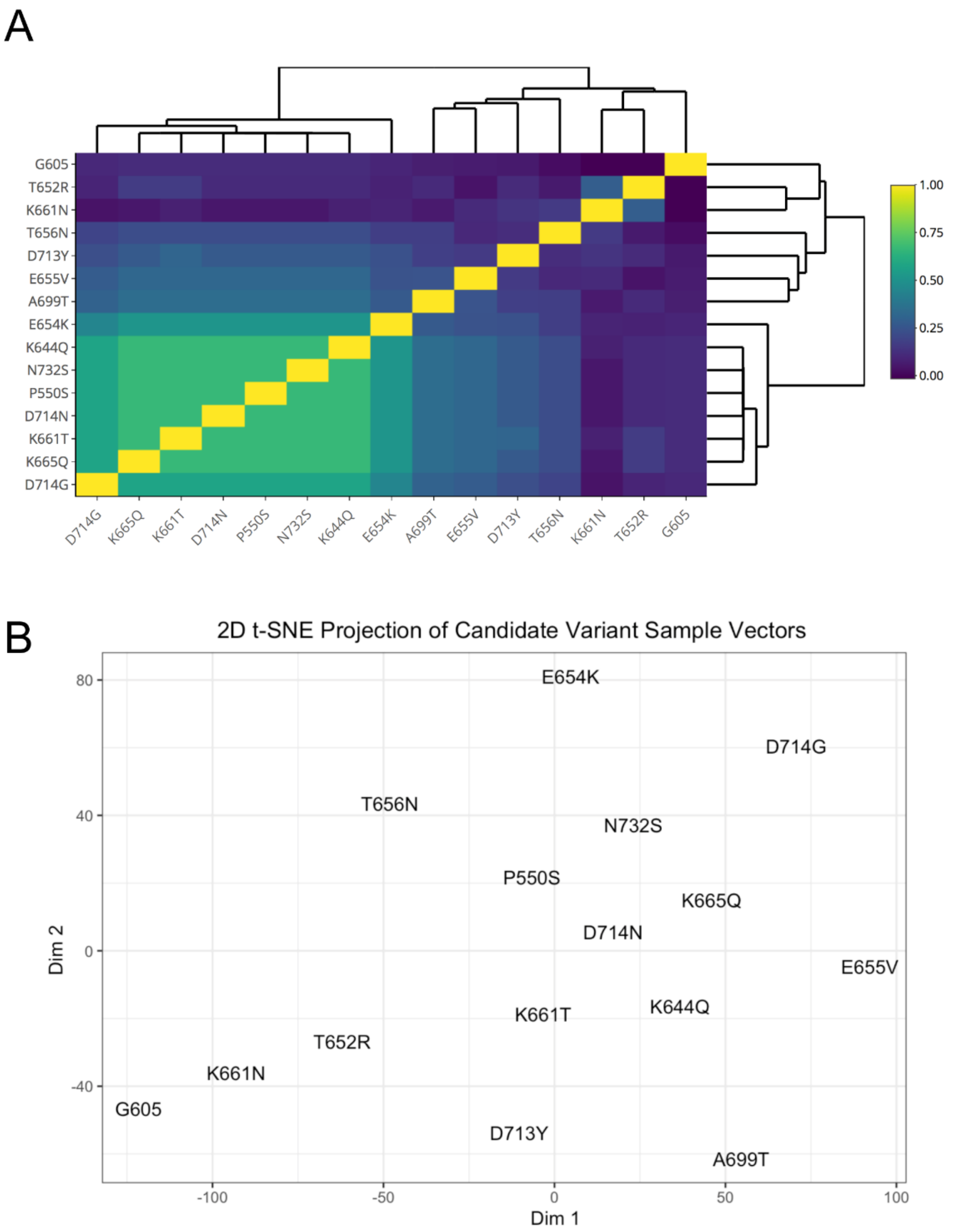

Supplementary Fig. 3. Visual analyses of polymorphism within the Pfs230D1 domain boundaries using a binary representation (presence/absence) of variant minor alleles from 2,618 *P. falciparum* samples obtained from the MalariaGEN Pf3k project pilot release 5. (A) Hierarchical heatmap of Pearson moment correlation values (low-to-high *r* values colored on a blue-to-yellow scale) for candidate missense variants in Pfs230D1. The cluster of variants near the bottom left in aqua green (K665Q, K661T, N732S, P550S, D714N and K644Q) share the highest pairwise correlations across samples (r = 0.67). (B). Two-dimensional t-SNE projection of candidate missense variants in Pfs230D1. Labels plotted closer to each other have distributions of alleles that are more similar across samples compared to those farther away. The same polymorphism cluster from (A) can be seen to form near the origin (0,0). The minor allele frequency tends to increase for variants in the plot as their distance from the origin (0,0) increases.

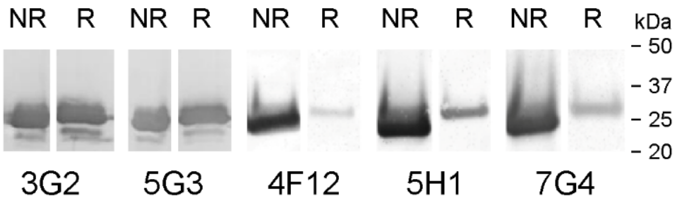

Supplementary Fig. 4. Western blot analysis using Pfs230D1-specific mAbs blotted against recombinant Pfs230D1 under non-reducing (NR) or reducing (R) conditions. MAbs 3G2 and 5G3 recognized linear epitopes within the N-terminal region of Pfs230D1 (amino acid residues 542 – 592) while mAbs 5H1 and 7G4 recognized reduction sensitive or conformation dependent epitopes. MAb 4F12 has been previously reported to recognize a conformation dependent epitope (1).

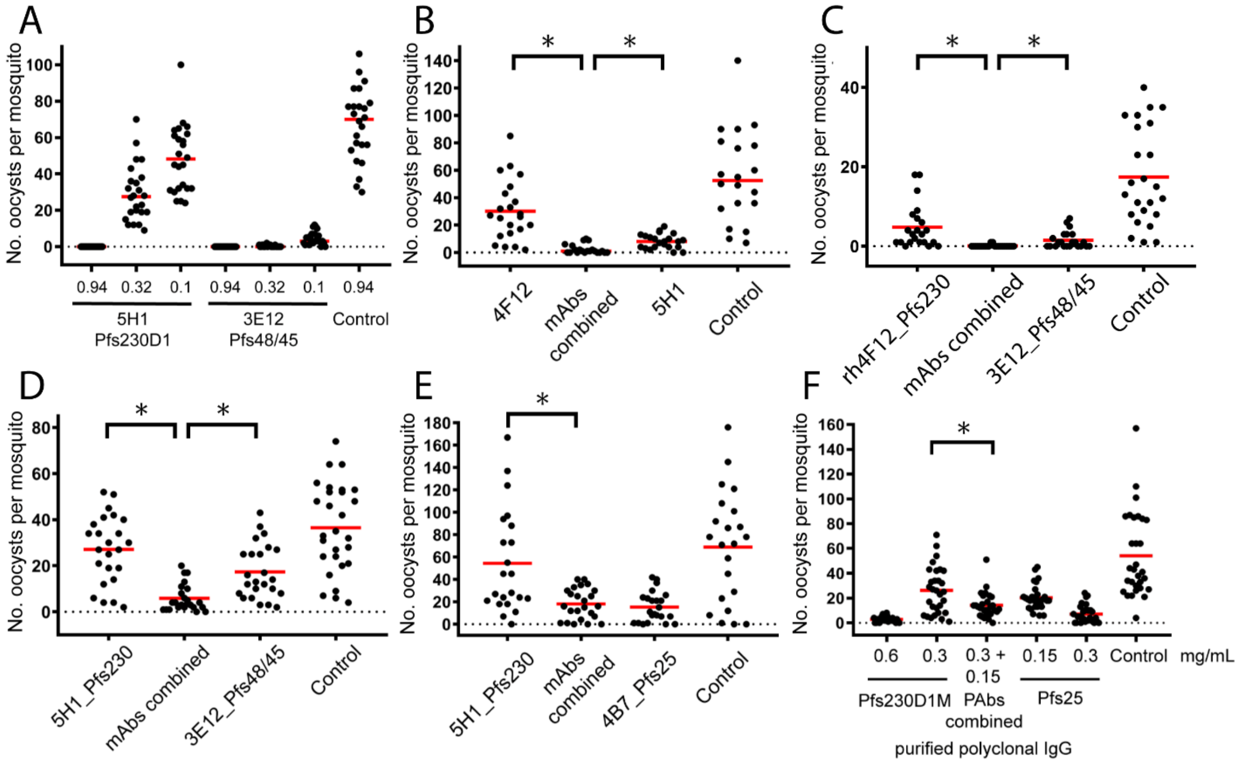

Supplementary Fig. 5. Assessment of functional activity of different mAbs following a titration and combinations of MAbs or pAbs against sexual stage parasites or zygotes/ookinetes. (A) Titration of TR activity of Pfs230D1-specific or Pfs48/45-specific mAbs in the standard membrane feeding assay (SMFA). The average inhibitions of oocyst density following titration of 5H1 were 100%, 56.3%, and 28.0%. For 3E12 the average inhibitions were 100%, 99.4%, and 93.2% using mAb 48F8 as a control. (B) Feeding assay results using Pfs230D1-specific mAbs 4F12 and 5H1, alone and combined. The average inhibitions of oocyst density by the Pfs230D1-specific mAbs alone and in combination are 46.1%, 95.7%, 85.4%, and 0% using 48F8 as a control at IgG concentrations of 0.94, 0.94+0.34, 0.34 and 1.29 mg/mL, respectively. (C) Combining Pfs230D1-specific mAb rh4F12 *("rh4F12_Pfs230")* and Pfs48/45-specific mAb 3E12 (*"3E12_Pfs48/45"*) shows enhanced TB activity (*"mAbs combined"*) compared to each set of mAbs alone (*, $p<0.001$). The average inhibition of oocyst density was 72.3%, 99.5% and 91.1% against the control mAb 48F8 (0%) at IgG concentrations: 0.63, 0.63+0.04, 0.04 and 0.94 mg/mL, respectively. (D) Feeding assay results using Pfs230D1-specific mAb 5H1 and Pfs48/45-specific mAb 3E12. The inhibitions of oocyst density by the two mAbs alone and in combination are 25.7%, 84.0%, 52.6%, and 0% (48F8 as the control) compared to the control at IgG concentrations of 0.2, 0.2+0.038, 0.038 and 0.3 mg/mL, respectively. (E) Evaluation of transmission blocking activity of Pfs230D1-specific and Pfs25-specific mAbs against these sexual stage proteins alone or in combination. The inhibitions of oocyst density were 21%, 73.7%, 77.7%, and 0% compared to the control at IgG concentrations of 0.25, 0.25+0.1, 0.1 and 0.35 mg/mL. (F) Evaluation of TB activity of Pfs230D1-specific and Pfs25-specific pAbs against these proteins alone or in combination. The inhibitions of oocyst density were 94.7%, 51.8%, 74.0%, 62.5%, and 87.1% in comparison to the adjuvant only control. Bars with an asterisk comparing each condition alone or in combination indicate statistical comparisons were significantly different ($p<0.001$). All assays included human complement.

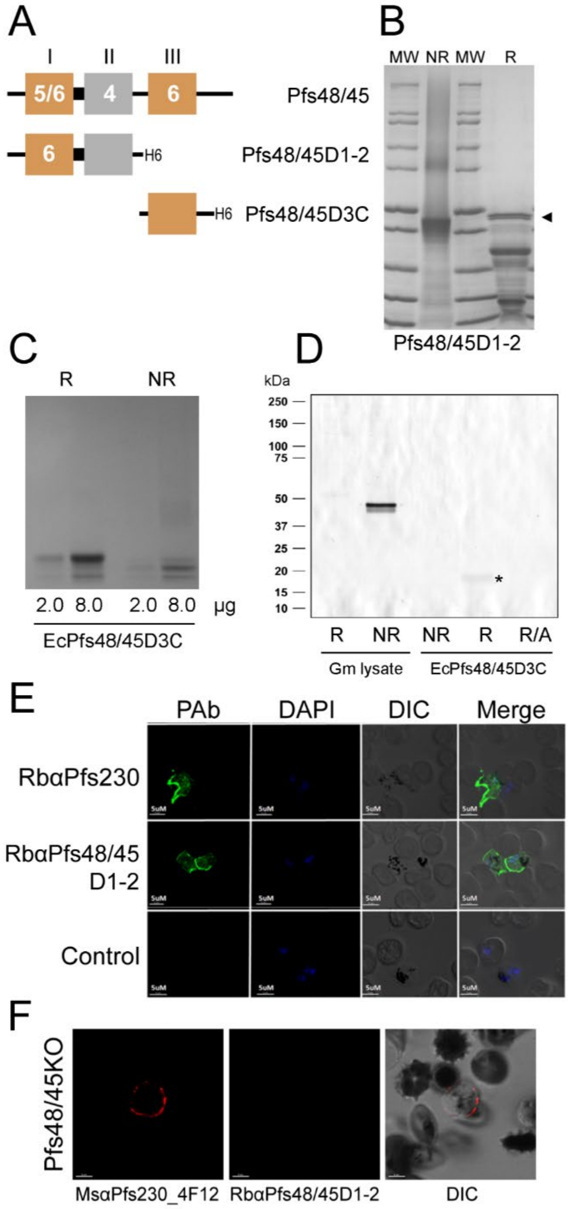

Supplementary Fig. 6. Recombinant protein expression and characterization of Pfs48/45. (A) The 6-cysteine rich Pfs48/45 protein schematic is shown in addition to two recombinant forms produced in *P. pastoris*, Pfs48/45D1-2 or in E. coli, Pfs48/45D3C each with a his-tag (H6). (B) SDS-PAGE gel Coomassie blue stained analysis of purified Pfs48/45D1-2 under reducing (R) and non-reducing (NR) conditions. Arrow points to Pfs48/45D1-2. (C) SDS-PAGE gel Coomassie blue stained analysis of purified EcPfs48/45D3C under reducing (R) and non-reducing (NR) conditions and low (2.0 ug) and high (8.0 ug) loadings. (D) Western blot analysis of Pfs48/45D3C with Pfs48/45-specific mAb 3E12 showing reactivity with parasite ("Gm") lysate and in-gel refolded EcPfs48/45D3C (asterisk). (E) Confocal microscopy shows live staining of *P. falciparum* gametes with rabbit antisera raised against Pfs48/45D1-2 ("RbαPfs48/45D1-2"). Rabbit Pfs230D1-specific antisera ("RbαPfs230") was a positive control and adjuvant alone antisera were included as a negative control. R/A is reduced and alkylated protein. Error bars represent 5 µm (D) and 2 µm (E), respectively.

SUPPLEMENTARY TABLES

Supplementary Table 1. Data collection and refinement statistics.

	Pfs230D1M-4F12 Fab Complex
**Data collection**	
Space group	$P2_12_12$
Cell dimensions	
a, *b*, *c* (Å)	79.954, 191.334, 41.198
Resolution (Å)	24.05 – 2.39
I / σI	17.16 (3.86)
Completeness (%)	98.4 (91.9)
$CC_{1/2}$	99.9 (95.7)
Redundancy	4.79
R-merge (%)[a]	5.1 (32.1)
**Refinement**	
Resolution (Å)	24.05 – 2.39
No. of reflections	25665
R_{work} / R_{free} (%)	22.92/27.71
No. of atoms	
Protein	4208
Ligand (Additives)	96
Water	60
B-factors (Å^2)	
Chain A	73.81
Chain B	67.86
Chain C	90.23
Ligand (Additives)	60.27
Water	57.93
Root mean square deviations	
Bond lengths (Å)	0.005
Bond angles (°)	0.738

*Values in parentheses are for highest-resolution shell.

[a] R-merge(I) = $\sum_{hkl}(\sum_i |I_i(hkl) - \langle I(hkl)\rangle| / \sum_{hkl}\sum_i I_i(hkl)$, where $I_i(hkl)$ is the intensity of the i-th observation of a reflection with indices (hkl), including those of its symmetry mates, and <I(hkl)> is the corresponding average intensity for all i measurements.

Supplementary Table 2. Standard membrane feeding assay results for positive and negative controls.

Antigen	mAb*/ isotype	Exp No.	TBA with human complement				
			Inf/Diss†	Avg No. oocysts	Median No. oocysts (range)	% reduction oocysts	% reduction prevalence
Pfs25	4B7	1	20/20	6.6	6 (1 – 15)	90.4	0
	IgG2a	2	13/20	3.6	1.5 (0 – 17)	86.0	35
		3	15/20	4.2	3 (0 – 12)	90.3	25
		5	18/20	10.1	8 (0 – 29)	82.0	10
		6	19/21	6.9	4 (0 – 22)	89.4	9.5
		7	15/20	2.5	1.5 (0 – 7)	95.1	25
		8	15/20	9.9	7 (0 – 35)	87.7	25
Control^	48F8	1	20/20	71.2	76.7 (16 – 110)	-	-
	IgG1	2	20/20	25.4	24 (4 – 91)	-	-
		3	18/20	26.6	21.5 (0 – 88)	-	-
		4	20/20	99.9	109 (5 – 189)	-	-
		5	20/20	68.5	68.5 (6 – 135)	-	-
		6	22/22	62.4	71 (9 – 97)	-	-
	PBS	7	20/20	50.7	52 (7 – 115)	-	-
	hIgG	8	20/20	51.5	47 (5 – 116)	-	-

*Pfs25 specific mAb 4B7 is shown as a positive assay control using a fixed number of ELISA Units (3250EU) which equates to ~0.5 mg/mL IgG; mAbs were tested at ~1.0 mg/mL IgG except for Exp. 8 assessed 0.6 mg/mL; ^Control: mAb 48F8 recognizes *P. yoelii* P140/RON4.
†Abbreviations: Inf., infected; Diss., dissected; h, human

REFERENCE

1. MacDonald NJ, et al. (2016) Structural and Immunological Characterization of Recombinant 6-Cysteine Domains of the *Plasmodium falciparum* Sexual Stage Protein Pfs230. J Biol Chem 291(38):19913-19922.
