# Supplementary material for: Structure and function of a malaria transmission blocking vaccine targeting Pfs230 and Pfs230-Pfs48/45 proteins
Source: Commun Biol. 2020 Jul 24;3:395. doi: 10.1038/s42003-020-01123-9 (PMC7381611; doi:10.1038/s42003-020-01123-9)
Supplement: Supplementary file 4 — Description of Additional Supplementary Files [file 42003_2020_1123_MOESM4_ESM.pdf]

## Description of Additional Supplementary Files

**File Name**: **Supplementary Data 1**

**Description**: Singh et al Supplemental Raw Data ELISA Files, Legend: Excel file contains the plate absorbance measurements and appropriate labels for the graphical images presented in Figures 1B and 4A. Additional data for technical replicates not plotted are included

**File Name**: **Supplementary Data 2**

**Description**: Singh et al Supplemental Raw Data SMFA Files, Legend: Excel file contains the oocysts counts observed for the various test conditions in the ex vivo standard membrane feeding assay that are plotted in Figure 4B -D.
