# Supplementary material for: Structure and function of a malaria transmission blocking vaccine targeting Pfs230 and Pfs230-Pfs48/45 proteins
Source: Commun Biol. 2020 Jul 24;3:395. doi: 10.1038/s42003-020-01123-9 (PMC7381611; doi:10.1038/s42003-020-01123-9)
Supplement: Supplementary file 6 — Reporting Summary [file 42003_2020_1123_MOESM6_ESM.pdf]

nature research

Corresponding author(s): David L. Narum

Last updated by author(s): Jun 30, 2020

Reporting Summary

Nature Research wishes to improve the reproducibility of the work that we publish. This form provides structure for consistency and transparency in reporting. For further information on Nature Research policies, see Authors & Referees and the Editorial Policy Checklist.

Statistics

For all statistical analyses, confirm that the following items are present in the figure legend, table legend, main text, or Methods section.

n/a	Confirmed	
☐	☒	The exact sample size (*n*) for each experimental group/condition, given as a discrete number and unit of measurement
☐	☒	A statement on whether measurements were taken from distinct samples or whether the same sample was measured repeatedly
☐	☒	The statistical test(s) used AND whether they are one- or two-sided<br>*Only common tests should be described solely by name; describe more complex techniques in the Methods section.*
☐	☒	A description of all covariates tested
☐	☒	A description of any assumptions or corrections, such as tests of normality and adjustment for multiple comparisons
☐	☒	A full description of the statistical parameters including central tendency (e.g. means) or other basic estimates (e.g. regression coefficient) AND variation (e.g. standard deviation) or associated estimates of uncertainty (e.g. confidence intervals)
☐	☒	For null hypothesis testing, the test statistic (e.g. *F*, *t*, *r*) with confidence intervals, effect sizes, degrees of freedom and *P* value noted<br>*Give P values as exact values whenever suitable.*
☒	☐	For Bayesian analysis, information on the choice of priors and Markov chain Monte Carlo settings
☒	☐	For hierarchical and complex designs, identification of the appropriate level for tests and full reporting of outcomes
☐	☒	Estimates of effect sizes (e.g. Cohen's *d*, Pearson's *r*), indicating how they were calculated

Our web collection on statistics for biologists contains articles on many of the points above.

Software and code

Policy information about availability of computer code

Data collection	No software was used for data collection in this study.
Data analysis	Bcftools ver. 1.9, UCSF Chimera ver. 1.13.1

For manuscripts utilizing custom algorithms or software that are central to the research but not yet described in published literature, software must be made available to editors/reviewers. We strongly encourage code deposition in a community repository (e.g. GitHub). See the Nature Research guidelines for submitting code & software for further information.

Data

Policy information about availability of data

All manuscripts must include a data availability statement. This statement should provide the following information, where applicable:

- Accession codes, unique identifiers, or web links for publicly available datasets
- A list of figures that have associated raw data
- A description of any restrictions on data availability

The datasets generated during and/or analyzed during the current study are available from the corresponding author on reasonable request.

Field-specific reporting

Please select the one below that is the best fit for your research. If you are not sure, read the appropriate sections before making your selection.

☒ Life sciences ☐ Behavioural & social sciences ☐ Ecological, evolutionary & environmental sciences

For a reference copy of the document with all sections, see nature.com/documents/nr-reporting-summary-flat.pdf

Life sciences study design

All studies must disclose on these points even when the disclosure is negative.

Sample size	N/A
Data exclusions	No data were excluded from the study analyses.
Replication	All studies were replicated and replicate findings are included in the supplemental information appendices.
Randomization	N/A
Blinding	N/A

Reporting for specific materials, systems and methods

We require information from authors about some types of materials, experimental systems and methods used in many studies. Here, indicate whether each material, system or method listed is relevant to your study. If you are not sure if a list item applies to your research, read the appropriate section before selecting a response.

Materials & experimental systems

n/a	Involved in the study	
☐	☒	Antibodies
☐	☒	Eukaryotic cell lines
☒	☐	Palaeontology
☐	☒	Animals and other organisms
☒	☐	Human research participants
☒	☐	Clinical data

Methods

n/a	Involved in the study	
☒	☐	ChIP-seq
☒	☐	Flow cytometry
☒	☐	MRI-based neuroimaging

Antibodies

Antibodies used	Murine cell line 4F12, 4F12(3B6), 5H1, 3G2, 5G3, 7G4, 48F8, 4B7; Anti-Mouse IgG (H+L) Antibody, Rabbit and Human Serum Adsorbed and Phosphatase-Labeled, Seracare Cat. # 5220-0312; Anti-mouse IgG (gamma) antibody, human serum adsorbed and peroxidase-labeled, Seracare Cat. # 5220-0339; Goat anti-Rabbit IgG (H+L) Highly Cross-Adsorbed Secondary Antibody, Alexa Fluor 488, ThermoFisher Scientific A-11034; Goat anti-Rabbit IgG (H+L) Highly Cross-Adsorbed Secondary Antibody, Alexa Fluor 594, ThermoFisher Scientific A-11037; Goat anti-Mouse IgG (H+L) Highly Cross-Adsorbed Secondary Antibody, Alexa Fluor 488, ThermoFisher Scientific A-11029; Goat anti-Mouse IgG (H+L) Highly Cross-Adsorbed Secondary Antibody, Alexa Fluor 594, ThermoFisher Scientific A-11032.
Validation	Murine cell line: 4F12 and 4F12(3B6), IFA, Western blot, standard membrane feeding assay (SMFA), isotyping, ELISA, cDNA sequencing; murine cell lines (MacDonald et al., J. Biol. Chem., 2016 291:19913-19922 and data provided in manuscript); isotyping 5H1, 3G2, 5G3, 7G4, IFA, Western blot, SMFA, ELISA (data provided in manuscript); murine cell line: 3E12, Western blot, IFA, isotyping; murine cell line (ATCC documentation and data provided in manuscript; 48F8, murine mAb specific for PyP140/RON4 (Narum et. al., Infect. & Immun. 2008, 76:4876-4882); 4B7, murine mAb specific to Pfs25 (Barr, P. J, et al. J. Exp. Med. 174 (1991): 1203-1208)

Eukaryotic cell lines

Policy information about cell lines

Cell line source(s)	HEK293F from Fisher Scientific
Authentication	Cells were not authenticated
Mycoplasma contamination	Cells were not tested for mycoplasm
Commonly misidentified lines (See ICLAC register)	No commonly misidentified cell lines were used

Animals and other organisms

Policy information about studies involving animals; ARRIVE guidelines recommended for reporting animal research

Laboratory animals	3 female BALB/c mice, 6 - 8 weeks of age were used for hybridoma development and female New Zealand White rabbits, 12 - 14 weeks of age.
Wild animals	The study did not involve wild animals.
Field-collected samples	The study did not involve collection of samples from the field.
Ethics oversight	All animals used for this project were approved by the National Institute of Allergy and Infectious Diseases, Division of Intramural Research, Animal Care and Use Committee, protocol: ASP LMIV 1E at the National Institutes of Health which is AAALAC accredited and OLAW assured. The NIAID DIR Animal Care and Use Program acknowledges and accepts responsibility for the care and use of animals involved in activities covered by the NIH IRP’s PHS Assurance #A4149-01.

Note that full information on the approval of the study protocol must also be provided in the manuscript.
